# Targeting the tumor-stromal-immune cell axis

**DOI:** 10.18632/oncoscience.190

**Published:** 2015-08-11

**Authors:** Murali Gururajan, Sajni Josson, Leland W.K. Chung

**Affiliations:** Uro-oncology Research Program, Cedars-Sinai Medical Center, Los Angeles, CA, USA; Bristol-Myers Squibb, Princeton, NJ, USA; Uro-oncology Research Program, Cedars-Sinai Medical Center, Los Angeles, CA, USA

**Keywords:** Immuno oncology, tumor microenvironment, cancer associated fibroblasts, microRNAs, myeloid cells

**Figure 1:**
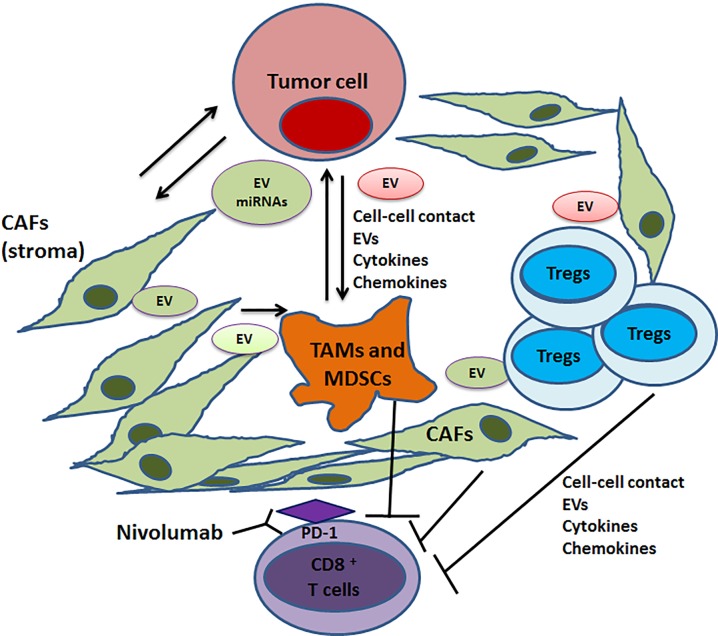
Barriers in the tumor microenvironment that dampens anti-tumor immunity Tumor cell-stromal fibroblast interactions lead to the secretion of soluble and insoluble factors and the release of extracellular vesicles carrying miRNAs and proteins, resulting in recruitment of immune-suppressive cells and macrophages in the TME. The predominant immune-suppressive cell types include TAMs, MDSCs and Tregs which prevent intratumoral trafficking of CD8+ T cells and dampen anti-tumor immunity. Nivolumab therapy (anti-PD1 antibody) rescues exhausted T cells but the efficacy is hindered by stromal barrier in some tumor types that prevent intratumoral trafficking of CD8+ T cells.

Cancer associated fibroblasts (CAFs) in the tumor microenvironment (TME) play a critical role in tumor progression. We and others have demonstrated that stromal fibroblasts can transform adjacent epithelial cancer cells *in vivo*. Cancer cells and stromal cells interact through physical contact, mediated by soluble factors, insoluble extracellular matrices (ECMs) or extracellular vesicles (EVs) including the “exosomes” and “oncosomes” [1-3]. EVs released in the TME act as cargo and are loaded with cellular DNA, RNA and protein. We demonstrated the transfer of small RNAs called microRNAs (miRNAs) through EVs from the CAFs into the adjacent epithelia, resulting in explosive tumor growth in preclinical mouse models [4]. In addition, the miRNAs promote epithelial to mesenchymal transition, a process that drives cell migration, invasion, and ultimately homing to bone and soft tissues [5]. Specifically, the miRNA members (miR-409, miR-379 and miR-154*) within the delta-like 1 homolog-deiodinase, iodothyronine 3 (DLK1-DIO3) imprinted region located on human chromosome 14 had tumor-inductive effects *in vitro* and in prostate cancer xenograft models. DLK1-DIO3 miRNAs have been shown to be essential for embryogenesis and induced pluripotent stem cell formation, and possibly could be hijacked during tumorigenesis, tumor-stroma interaction and cancer metastasis. Cancer cells activate embryonic programs and pathways that partially maintain stem cell identity, often referred as “stemness.” We demonstrated that the DLK1-DIO3 cluster miRNAs derived from EVs of CAFs promote EMT and stemness of adjacent epithelial cells *in vitro* and *in vivo* [4, 5]. However, most published studies have focused on the study of tumor cell-CAF interactions using xenograft models. These studies provide valuable information on the mechanistic basis of tumor/CAF interactions and molecular targets that mediate these interactions including the role of EVs and EV-loaded miRNAs in the TME. However, the impact of tumor-CAF interactions on immune cells, a major cell type in TME mediating anti-tumor immunity, requires studies in immune-competent preclinical tumor models. The TME is enriched with immune cells including myeloid cell types like tumor-associated macrophages (TAMs) and myeloid derived suppressor cells (MDSCs). These immune-suppressive cell types inhibit T cell mediated anti-tumor immunity through cell-cell interactions and extracellular factors including EVs (Figure 1). The consequence of EV-mediated transfer of miRNAs and soluble factors between tumor, CAFs and myeloid cells is not known. Given the recent surge in FDA approved therapeutic modalities that target immune cells in TME, future studies for understanding tumor-CAF interactions and the impact of EVs on immune cells in TME are imperative.

Therapeutic targeting of CAFs with an anti-fibrotic agent, tranilast, led to reduced infiltration of immune suppressive cell types, including regulatory T cells and MDSCs in the TME in preclinical immune-competent mouse models of lung cancer, melanoma and lymphoma [6]. However, the critical mediators of these therapeutic responses have not been identified. It is possible that extracellular vesicle mediated transfer of miRNAs and proteins promote infiltration of immune-suppressive cell types in TME and that targeting CAF leads to reduced infiltration of such cell types, and restoration of anti-tumor immune responses by tumor-associated antigen-specific CD8+ T cells. Thus, CAF-targeted therapy enhanced tumor immune surveillance and synergistically promoted systemic anti-tumor immune responses in combination with dendritic cell-based vaccines only in mice with an intact immune system [6]. These data in aggregate indicate that systemic anti-tumor immune responses are better elicited when barriers in TME are disrupted, including CAF-targeted therapy.

We demonstrated previously that radiation-induced miRNAs in prostate cancer cells confer resistance to therapy and that targeting miRNAs restored radiation sensitivity. Radiation therapy also promotes/enhances anti-tumor immunity through cell death and release of tumor antigens in TME. Whether miRNAs mediate radiation-induced modulation of TME is not well understood. It will be interesting to explore the effects of CAF targeted therapies and/or low dose radiation for enhancing the anti-tumor efficacy of checkpoint inhibitors. Highly successful FDA-approved antibody-based checkpoint inhibitors like Ipilimumab and Nivolumab have had remarkable success in the clinic for treating melanoma and lung cancer patients but only limited success in pancreatic, glioblastoma, and bone metastatic prostate cancers due to barriers created by the presence of significant stromal components including CAFs and macrophages that prevent intra-tumoral entry of anti-tumor cytotoxic T cells [7]. Therefore, studies on stroma (CAFs and TAMs) present an exciting prospect (Figure 1), as such studies will lead to the identification of novel therapeutic modalities to augment/facilitate the use of checkpoint inhibitors and other cancer immunotherapy/immuno-oncology drugs in the clinic. Unraveling the elements that mediate tumor-stromal-immune cell interactions, including cell surface molecules and extracellular soluble and insoluble factors like cytokines, chemokines, ECMs and EVs and the cargo associated with these EVs, is imperative for the development of novel therapies that target TME. Exosomes and oncosomes also serve as useful biomarkers in many types of cancers including lethal pancreatic and bone metastatic prostate cancer [5, 8]. Targeting the tumor-stromal-immune cell axis in TME by small molecule based drugs and biologics holds great promise as a future cancer therapy.
